# Designing structured postgraduate training programs using agile methods

**DOI:** 10.3205/zma001720

**Published:** 2024-11-15

**Authors:** Luke S. Hopf, Katja Doerry, Ann-Kristin Grzybowski, Katharina Hermann, Jessika Johannsen, Aloisa Stadlhofer, Michael Krumm, Frauke van der Meer, Kevin Paul, Nuno Ramos Leal, Ania C. Muntau, Søren W. Gersting

**Affiliations:** 1 University Medical Center Hamburg-Eppendorf, Children’s Hospital, Department of Pediatrics, Hamburg, Germany; 2 Pediatrician’s office Weidenallee, Hamburg, Germany; 3 Fielmann Group AG, Hamburg, Germany; 4 University Medical Center Hamburg-Eppendorf, Center of Obstetrics and Pediatrics, Division of Neonatology and Pediatric Critical Care Medicine, Hamburg, Germany; 5 University Medical Center Hamburg-Eppendorf, University Children’s Research, Hamburg, Germany

**Keywords:** postgraduate medical education (PGME), curriculum, agile, program development, competency-based education

## Abstract

Postgraduate medical education (PGME) is an essential part of medical education and increasingly shifts into focus of educational stakeholders. Structured postgraduate medical training programs are required in the U.S. through the “American Council for Graduate Medical Education” (ACGME) guidelines with their six core competencies as common program requirements. The basis for this development was provided in Germany with the implementation of the “Standard Framework for Postgraduate Medical Training” (Musterweiterbildungsordnung) issued by the German Medical Association (Bundesärztekammer). However, implementation has been gradual and program development is often conducted in a time consuming, lengthy and top-down approach without that trainee experiences or needs are being assessed or evaluated for their impact on successful medical training.

We demonstrate how application of agile working can enable rapid and efficient creation and implementation of a novel postgraduate training program. The postgraduate training program ped.tracks aims to achieve a high-quality, structured and reliable postgraduate training. Moreover, it provides the opportunity to select a priority on scientific or clinical education. The entire process from the first draft to full release of the program was completed within 8 months through agile working. Our team worked using agile working techniques, creating a trainee- customized and -centred program. We anticipate that the quantity of structured postgraduate training programs will significantly increase in Germany and Europe to improve training quality and employee satisfaction. Therefore, the use of agile methods for the creation and implementation of structured training programs represents a useful approach to support program directors rapidly and effectively in this effort.

## Abstract (de)

Die postgraduierte medizinische Ausbildung (PGME) ist ein wesentlicher Bestandteil der medizinischen Ausbildung und rückt zunehmend in den Fokus von Weiterbildungseinrichtungen und -befugten. Strukturierte postgraduierte medizinische Ausbildungsprogramme werden in den USA durch die Richtlinien des „American Council for Graduate Medical Education“ (ACGME) mit ihren sechs Kernkompetenzen vorgegeben. Die Grundlage für deren Entwicklung auch in Deutschland wurde mit der Einführung der Musterweiterbildungsordnung der Bundesärztekammer geschaffen. Die Umsetzung erfolgte in vielen Fällen schrittweise und die Entwicklung neuer Weiterbildungsprogramme wird häufig in einem zeitaufwändigen, langwierigen und hierarchisch verlaufenden Ansatz durchgeführt, ohne dass die Erfahrungen oder Bedürfnisse der Auszubildenden im Hinblick auf ihre Auswirkungen zu einer erfolgreichen medizinischen Ausbildung einbezogen werden.

Wir zeigen, wie die Nutzung agilen Arbeitens die schnelle und effiziente Erstellung und Implementierung eines neuartigen postgraduierten Ausbildungsprogramms ermöglicht. Das postgraduierte Ausbildungsprogramm ped.tracks zielt auf eine qualitativ hochwertige, strukturierte und zuverlässige postgraduierte Ausbildung ab. Darüber hinaus bietet es die Möglichkeit, einen Schwerpunkt auf die wissenschaftliche oder klinische Ausbildung zu legen. Der gesamte Prozess vom ersten Entwurf bis zur vollständigen Freigabe des Programms wurde innerhalb von 8 Monaten durch agiles Arbeiten abgeschlossen. Unser Team arbeitete mit agilen Arbeitstechniken und entwickelte ein auf die Auszubildenden zugeschnittenes Programm. Wir gehen davon aus, dass der Bedarf an strukturierten Weiterbildungsprogrammen in Deutschland und Europa deutlich zunehmen wird, um die Ausbildungsqualität und die Mitarbeitendenzufriedenheit zu verbessern. Der Einsatz agiler Methoden für die Erstellung und Umsetzung strukturierter Weiterbildungsprogramme stellt dabei einen sinnvollen Ansatz dar, um Weiterbildungsbefugte schnell und effektiv bei diesem Vorhaben zu unterstützen.

## 1. Introduction

Postgraduate medical training is getting increasing attention, considering the growing importance to qualify medical personnel, the rapid speed of advancements in medical sciences and the need for highly skilled healthcare professionals. The United States and Canada have been setting the standards for centralized guidance in their postgraduate training through e.g. Accreditation Council for Graduate Medical Education (ACGME) guidelines and the CanMEDs framework. Europe has been lacking homogeneous accreditation criteria and frameworks, even though the European Union specifies diplomas within the Union must be mutually recognized without further assessment [1]. On an international scale, the field of specialty training remains heterogeneous [2], [3]. Variations on continuing medical education exist on nearly every level from admission policy to licensing. Despite efforts of organizations like the “European Union of Medical Specialists”, outcomes of training programs in the EU might differ [4], [5]. This leads to a heterogeneous postgraduate training quality, which is still considered to be equal on paper. This is problematic for a number of reasons and may cause discordant educational quality as well as impaired patient safety. 

Even on a national scale within Germany, qualification specifications may differ for clinical specialist training. Specialist qualification usually involves postgraduate training for 5-6 years and educational requirements are specified by the 17 state medical associations. Postgraduate medical training is not tied to academic centers. Training is often unstructured and primarily the responsibility of the trainee [6]. This can lead to heterogeneous training outcomes even within the same department and lead to training outcomes being left open to subjective aspects like luck, sympathy from the department head or decision-makers and self-sacrifice, even leaving the educational success open to potential systematic discrimination. 

To tackle these problems, the German Medical Association published a “Standard Framework for Postgraduate Medical Training” (Musterweiterbildungsordnung), which provides a competency-based template for all State Medical Associations aiming to revise their specialty-training qualifications. In general, two major competency levels are distinguished, which are firstly cognitive and knowledge-based and secondly skill-based competencies. The standard framework defines general competencies every physician needs to acquire, like basics of patient centred care, ethical, scientific, and legal knowledge only to name a few. Every specialty has defined competencies that the learner must achieve in order to be granted the specialty title. In paediatrics, these competencies include developmental and social paediatrics, paediatric emergency and intensive care, neonatal diseases, and adolescent medicine. In addition, the novel framework requires program directors to add a structured training outline to receive authorization and allow for postgraduate training under their supervision [7]. The advantages of competency based postgraduate medical education (PGME) have been explored intensely [8]. They can define outcomes, provide for formative and summative assessment, and allow learning for mastery. Competency based medical education has also been an increasingly important aspect of medical education and curriculum development [9].

So far, many postgraduate medical training programs have not seized the opportunity to innovate yet. In a survey only 24-40% of trainees reported that structured postgraduate training was adhered to [10], [11]. In general, the lack of structured, reliable, and competency-oriented training plans results in lower workplace satisfaction and education quality. In 2009 the German Medical Association started to evaluate training programs in Germany. However, the evaluations have been in the responsibility of the State Medical Associations and therefore the frequency and quality have been varying depending on the local medical associations. The last national evaluation occurred in 2011. A main aspect criticized in this survey was the lack of communication of education progress and rotation plans [12]. Therefore, a clearly structured educational track with preset feedback opportunities seems to be an attractive measure for trainees to plan their educational path and reflect on their knowledge and personal growth during training.

The demand and need for novel and structured training programs are high, but the creation and implementation during routine clinical duties remain challenging for department heads and stakeholders. Program development is often considered time consuming, lengthy and resource intensive. Therefore, programs are often implemented using a top-down approach without assessing or realistically evaluating trainees’ experiences or needs, leading to a decline in innovation [13], [14], [15]. 

The need for rapid, streamlined, and user-friendly innovation has also been a major issue in software development. Here, this need was met through creation and widespread adoption of agile working methods since the early 2000s with predominantly positive reports [16]. Agile working is a flexible and collaborative approach which promotes flexibility, iterative processes, and customer-orientation. These methods have been utilized in the context of the rapidly growing internet software industry to focus on process-oriented methods and enable developers to adapt quickly in a fast-changing market. These agile working theories have been transferred to other fields of project management for example in risk management, medical device companies and the pharmaceutical industry [17], [18], [19]. Here we demonstrate how we utilized agile working to create and implement an innovative training program. With this contribution, we aim to provide guidance to other departments and encourage them to develop new and structured training programs in a simple and easy-to-use manner.

The program titled ped.tracks aims to focus on individual career interests as well as ensuring a structured and reflective postgraduate training for all trainees enrolled in the department by offering a set of clinical tracks, structured rotations, mentoring and additional qualification training. 

## 2. Methods

### Agile working

The foundation of agile working, following the agile manifesto, are 4 values and 12 principles [20]. In general, it emphasizes human interaction and rapid learning cycles as basis for success when writing software. The 4 values are “Individuals and interactions over processes and tools”, “Working software over comprehensive documentation”, “Customer collaboration over contract negotiation” and “Responding to change over following a plan” [20]. The 12 principles can be summarized as promoting customer satisfaction, embracing changes, delivering working software regularly, endorsing interaction of working-staff and software developers, empowering motivated individuals, encouraging face-to-face meetings, measuring the success of progress through working software, maintaining a constant pace of development, promoting simplicity, supporting self-organization and promoting team reflection regularly [20]. 

While the agile manifesto has no prescribed processes or events, there are various frameworks that help put the agile values and principles into action. A very prominent framework is Scrum [21]. Regardless of which framework is applied, there are generally similar activities, which are essential to agile frameworks and methodologies:

1. Create a shared understanding of what shall be achieved.
2. Discuss and plan work that needs to happen to come closer to what is wanted. Do this in the smallest possible steps to receive feedback as soon as possible.
3. Work on the discussed work packages.
4. Release the work results/increments into production/real operation.
5. Learn and adapt.

Preferably, customers, users, co-workers, stakeholders or affected people are included in all steps. In our case, this means that all relevant users and affected staff were involved, including physicians, senior physicians, teaching staff, rotational partners, chief residents, department heads of other clinics, as well as relevant institutions and regulatory bodies. 

Although agile working offers a lot of different approaches, frameworks and methodologies, the foundation of all agile learning and working is based on the importance of humans and their interactions [22]. It emphasizes the relationship and communality of team members [23]. 

Also “agile” is not the only framework that can be applied to such problems. Various other frameworks like Prince2 [https://www.prince2.com/de/prince2-methodology] as well as project management methods in general are possible ways to approach similar problems. Following Simon Wardley’s approach to use methods based on the evolution of solutions from “genesis” to “commodity” agile is in the context of creating something new as it is discussed here a promising approach as it focusses on reducing the costs of change and can handle challenges that often come with novel solutions [https://medium.com/wardleymaps]. 

Agile methodologies, particularly frameworks like Scrum, have been explored for managing projects in collaborative research initiatives, showcasing their extension beyond software development to other types of organizational and knowledge management processes [24]. The adoption of agile principles, such as through Scrum, promotes structured yet flexible planning and execution processes, like sprint planning, which can be advantageous in tackling scientific project challenges [25]. Agile management has also been noted for its positive impact on project performance, with its various aspects contributing to optimized project outcomes when proper leadership competencies are in place [26]. Additionally, while organizations find value in agile project management, challenges in scaling agile methods to larger projects have been identified, indicating an area of exploration for maximizing the benefits of agile in diverse project scopes [27].

### Formation of the group

**Figure 1:**
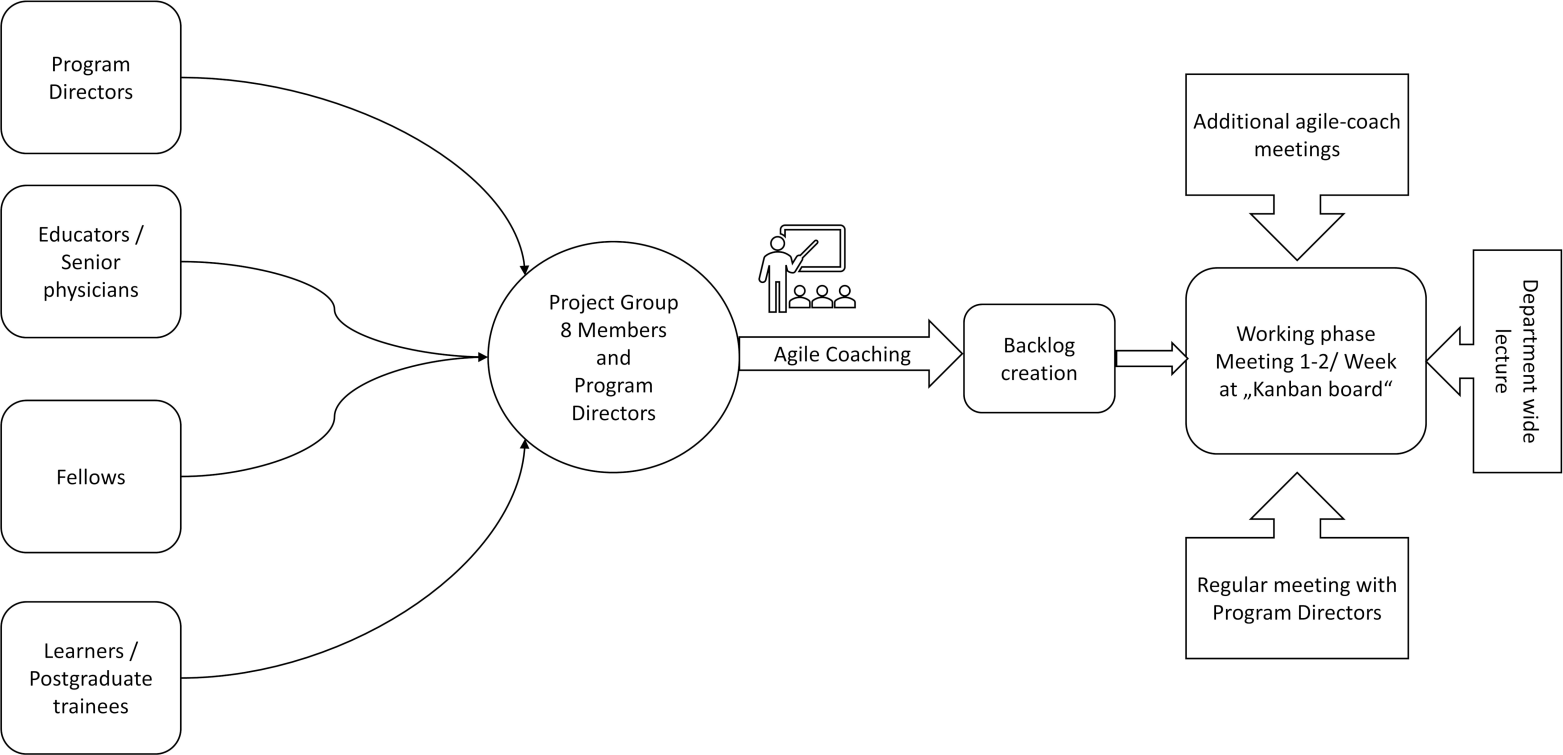
Group formation and working procedures

The program directors consisting of the director of the Children’s Clinic as well as the Director of the Children’s Research Centre selected 8 team members who were at various stages of residency training or shortly after completing postgraduate specialty training in the Department of Paediatrics. Additionally, the staff assistant senior physician was involved in the project and ensured that the program met guidelines for work hour limitations and other formalities. The team was selected to include a diverse group, representing all facets of the medical employees of the clinic. Individuals were selected based on special skills, knowledge, or involvement in further responsibilities in clinical management (e.g., rotation schedule-implementation). Selection criteria were also number of years in specialty training, high interest in educational training programs and postgraduate education in general, teamwork skills and high commitment to these additional duties. Members were informed individually in face-to-face meetings and later as a group by email. All members attended a 6-hour workshop for training in agile working. This included a general introduction to agile working, methods of using these aspects and practical examples (see figure 1 (Fig. 1)).

The program directors provided a basic framework on which the group could build upon. After basic training in agile working, a large whiteboard was placed in the lecture hall and a standard Kanban-Board set was provided. 

### Agile coach

The agile coach facilitates the initial training of the group in agile methods. Additionally, they are invited to board-sessions on regular intervals depending on the needs of the group. The term “agile coach” is neither protected nor well defined [28], [29]. The role of agile coaches is diverse and depends on the context they act in. It can be assumed that the person context fit is relevant to create an impact. Tasks vary broadly and range from “teaching and mentoring to helping the teams understand the agile methods, empowering them to ask relevant questions, discovering the knowledge already hidden in the team, resolving conflicts, and facilitating the overcoming human impediments in overall process improvement” [29].

### Backlog creation

The backlog contains all the individual tasks, which are necessary to complete the entire assignment. The initial backlog was compiled during an extensive brainstorming session at the beginning of the project in which all team members participated. Initial needs and limitations of the previous, unstructured training program were discussed, and improvements were debated. All assignments, which were thought to be necessary to complete the project, were collected, prioritized, and assigned to members of the group. The backlog was extended when deemed necessary during the course of the project. 

### Assignment progression 

**Figure 2:**
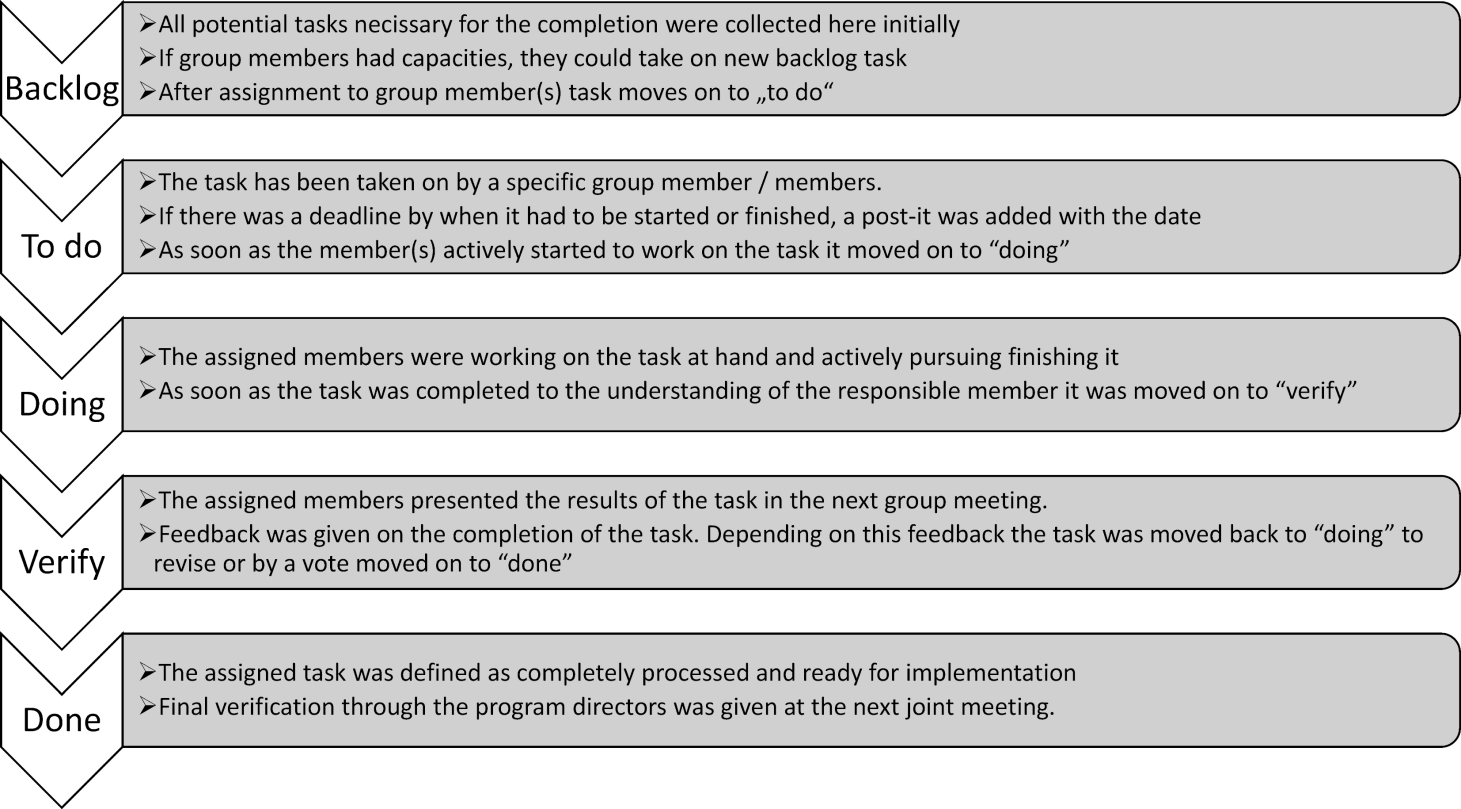
Assignment progression during agile working

A whiteboard was divided into different columns on which the assignments could be moved from left to right until completion. All assignments started on the left-hand side of the board in the “backlog” column. After initial assignment to a specific person or persons, it was moved to the second column, labeled “to do”. As soon as the assigned person/s were actively pursuing these assignments, it was moved to the third column, labeled “doing”. If the assignment was completed, it was placed in the fourth column labeled “verify”, which meant, this assignment was discussed in a team meeting and either placed back into the third column, if it was not complete or placed in the fifth column, labeled “done”, which signaled, this assignment was satisfactory to the entire group (see figure 2 (Fig. 2)).

### Decision finding 

Major decisions were discussed and decided at a Kanban-Meeting after sufficient discussion. At least four members had to be present to reach a decision. Decisions were re-visited if new aspects were introduced or members who were not part of the initial discussion had concerns. The program directors and the staff assistant senior physician could veto important decisions if e.g., financial, or formal guidelines were of concern. 

## 3. Results

### Agile working procedure 

In this first phase of the project, the group met twice a week for a Kanban-Meeting, Tuesday, and Thursday after the midday meeting. These meetings included reporting on progress, evaluating further steps, and distributing further assignments to individual members. These “Kanban-Meetings” lasted for about 10-15 minutes. Meetings were kept as short as possible because team members were involved in clinical duties that required a high level of group commitment. Meetings were often not supervised by a department head, so a high degree of trust and self-reliance was put in the group to find suitable solutions to problems. 

Meetings were held twice with the agile coach at the Kanban board to review difficulties and potential pitfalls. At least every two months, the program directors attended the Kanban-Meetings to review progress and evaluate the project. 

In the second phase, after about 4 months, Kanban-Meetings were reduced to once a week to accommodate the number of remaining tasks, which were usually more time-consuming or part of lengthy discussions with other departments or management, for example. 

Not all group members had to be present at each Kanban-Meeting. However, at least four members were necessary to discuss important decisions. 

The entire Department of Pediatrics was briefed on the project and interim results were presented in a department-wide lecture to gather further input and address the concerns of other staff. 

The creation of a text document, serving as a manual for the training outlines and the contents of the training program for the specialty training for Pediatric Medicine was a major milestone of the project. Upon completion of the manual, all relevant department heads were informed and asked for final authorization. The project was then announced and presented in a department-wide lecture. The implementation was set to the 1st January 2020. The entire project was completed within 8 months. Team members rated the meetings as highly effective, and the team dynamic was described as integrative and time-efficient. 

### ped.tracks – postgraduate specialty training program

**Figure 3:**
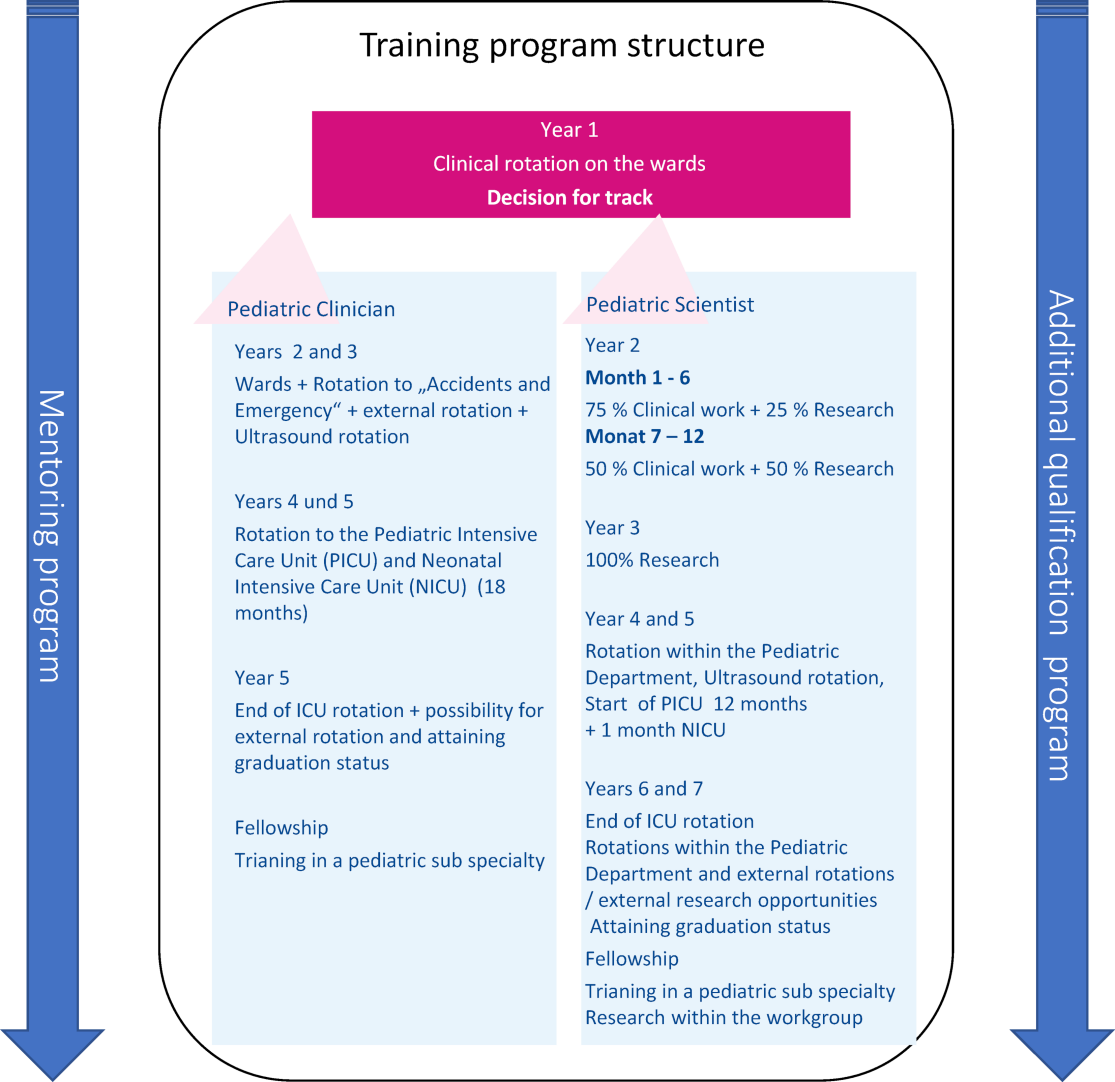
Training program structure ped.tracks

The main aim of this postgraduate training program was to help trainees achieve their individual career goals and produce medical experts at the highest professional level in the fields of healthcare, research and medical skills. In addition, this training program was designed to provide trainees with planning security and integrated feedback and reflection opportunities, which were lacking according to surveys and trainees’ own experiences. We achieved this by adding a structured schedule for training as well as fixed feedback sessions as part of the mentoring program (see figure 3 (Fig. 3)).

#### Competencies and skills for pediatric specialty training

The group discussed in depth whether to establish an individual competency-based curriculum to be completed during training. However, national, and international institutions (Hamburg Medical Association, European Academy for Pediatrics) provide these frameworks in detail. Therefore, the “Standard Framework for Postgraduate Medical Training” (Musterweiterbildungsordnung) from the Hamburg Medical Association as well as the “Training Requirements for the Specialty of Paediatrics” by the European Standards of Postgraduate Medical Specialist Training were used as a guideline for educational objectives during training and therefore no additional competency structure was implemented [30], [31].

#### Mentoring 

A general mentoring program as well as a research mentoring program were established to guide and support trainees during their specialty training. Each trainee chooses a mentor and meets with them once or twice a year. In addition, pediatric scientists chose a research mentor to further their scientific goals.

#### Training structure 

The first year begins with “basic training”. The trainee is assigned to the pediatric wards and works in shifts in the pediatric emergency department as well as late shifts and night shifts. After the first year of specialty training, the trainee can decide whether to apply either for the “pediatric clinician” or “pediatric scientist” track, depending on their individual career goals. This is discussed in meetings with the mentor, who is an experienced senior physician of the department and advises the trainee throughout his or her training. 

##### Pediatric clinician 

This track is designed for clinically oriented trainees who are likely to complete specialty training after five years. Early involvement in a pediatric sub-specialty department is recommended. The Pediatric Clinician track encourages graduates to pursue further specialty training (e.g., fellowships), apply for external senior consultant positions or join an outpatient pediatric practice. 

##### Pediatric scientist

This track is designed for scientifically oriented trainees who are likely to complete specialty training within 6-7 years. According to the need of the trainee, they are eligible for clinical research periods, starting in general with 25% in the second year of training and increasing up to 100% for an entire year in the third year of training. During this time, trainees will establish their own research project, learn research methods, acquire necessary qualifications, lay the basis for further research, and allow them to apply for research grants and other sources of funding for the future.

#### Rotations 

##### Mandatory rotations 

Each trainee must complete training in the ICU/NICU. Depending on the track chosen, trainees spend 18 months in the ICU/NICU for the pediatric clinician or 13 months for the pediatric scientist. In addition, a pediatric radiology rotation is mandatory regardless of track choice. The Pediatric specialty training comprises of specific quantities of common sonography-scans, which are performed during this rotation. These are acquired during a radiology rotation and late and night shifts during emergency care. 

##### Optional rotations 

Trainees can choose at least one out of seven specialty rotations during their postgraduate training. Rotations vary in duration and allow for further specialization tailored to individual career goals. Rotations are available at pediatric hematology & oncology, pediatric forensic and legal medicine, ambulatory pediatric practice, pediatric surgery, pediatric cardiology, and an external rotation to the Altona Children’s Hospital.

#### Additional qualification 

In addition to clinically focused specialty training for both tracks, trainees complete several additional modules, which are offered once a year in a workshop setting. Each trainee is encouraged to attend at least one workshop every year. The workshops address clinical study competency, research methodology development, funding and grant proposal writing, a genetic toolbox, senior physician teaching, healthcare economics, undergraduate instructor training, communication training and microteaching. 

#### Evaluation 

To adapt and continuously improve our training program, we aim to establish an extensive evaluation model, closely following guidelines to program evaluation [32], [33], [34].

The German Medical Association requires the recording of specialty training in a logbook provided by the association. The progress is regularly discussed with and signed by program directors.

## 4. Discussion

Curriculum and training program development is often part of a lengthy and complicated process and has usually been operated top-down without the trainee’s experience or needs being assessed or realistically evaluated [35], [36]. However, the fast pace of medical specialization, the shift to competency-based education and changing learner needs mandates rapid innovation tailoring to local needs and contexts. Software development and other industry-based development projects have been confronted with similar challenges in the past and established agile working methodology to increase effectivity of development processes. 

According to Fullan, the two interrelated reasons planning in educational change does not succeed, is failure to take local context and culture into account and too much emphasis on the planning relative to the action part [37]. Our own experience is also, that long term planning is complicated by life events like resignations, pregnancies, health issues, parental leave and other career opportunities. Agile working addresses these pitfalls by allowing for rapid development and speedy innovation while involving all relevant stakeholders and local context by early “end user engagement”, which has also shown to be effective and raise customer satisfaction in software development [38]. 

There is only little data available on the length or success rate of the design process in program or curriculum development. However, designing, implementing, and evaluating educational change continues to be one of the major challenges of educational stakeholders. There is an abundance of instructional design models described in the literature with differing popularity and complexity [39], [40]. Many have been criticized to not focus on the design process in itself [41]. 

In an anonymous post-process evaluation, the development team valued the flat hierarchy this process requires and the clear assignments of responsibilities. Also, they appreciated accomplishing small achievements continuously, which was a source of motivation. The high frequency of the meetings reminded members of their outstanding tasks regularly, preventing them to get delayed. Challenges for working agile were described as well. Firstly, the tight timeframe and managing the complexity of the project within many short sessions instead of a few long ones was demanding. Also, getting accustomed to working using agile methods for the first time was also described as challenging. Discussions were perceived as intermittently being unstructured, repetitive, and unproductive. Working within the short meetings on the spot also favors outspoken, confident, and assertive members of the team. Finally, at times low attendance of the team meetings due to shift work schedules slowed down the progress. 

The evaluation of ped.tracks is still ongoing and will be reported separately. In general, the trainees are principally satisfied with the program. One major improvement point mentioned by them is, that rotations are announced late and are perceived as surprising. Also, extracurricular activities as prescribed in the additional qualifications often clash with clinical duties and attendance can be an issue. 

By using the agile methods approach, we have successfully developed a specialized training program. It is efficient and integrative by working with the trainees and comparably easy to implement in any training program. However, this approach requires a high degree of commitment by the group and trust and conviction by the program directors to work effectively. A previously failed attempt to reform the program in a top down approach over the course of two years as well as the need to innovate in a growing competitive job market were the main drivers for this approach. 

 This project outline can be scaled, depending on the executing department. 

Considering this is a new approach to program development, evaluation and assessment will be conducted to investigate, if this is a promising method to establish training programs in clinical practice. 

Central lessons and tips for other departments learned by the team are that:

1. The composition of the team is essential and needs to represent all factions of the clinic.
2. All team members need to be intrinsically motivated and ready to take responsibility for the outcome.
3. The team needs to be properly trained in agile methods.
4. Stakeholders such as program directors need to fully trust and support the team and the process.
5. An external agile coach is imperative to support the team intermittently.

Concluding, we encourage other departments which need revision of their postgraduate training to utilize an agile working approach to implement a novel training program rapidly and effectively. Adjustments will have to be made, based on local factors. However, we believe that our approach is suitable and easy to implement within every department. 

## Authors’ ORCIDs

- Luke Hopf: [0000-0002-9436-335X]
- Katja Doerry: [0000-0001-8184-3592]
- Kevin Paul: [0000-0002-0998-4881]
- Ania C. Muntau: [0000-0002-2900-8378]
- Søren W. Gersting: [0000-0001-7482-4748]

## Competing interests

The authors declare that they have no competing interests. 

## References

[1] European Parliament, Council of the European Union (2005). Directive 2005/36/EC of the European Parliament and of the Council on the recognition of professional qualifications. Off J Eur Union.

[2] Wijnen-Meijer M, Burdick W, Alofs L, Burgers C, ten Cate O (2013). Stages and transitions in medical education around the world: clarifying structures and terminology. Med Teach.

[3] Weggemans MM, van Dijk B, van Dooijeweert B, Veenendaal AG, Ten Cate O (2017). The postgraduate medical education pathway: an international comparison. GMS J Med Educ.

[4] Cumming A, Ross M (2007). The Tuning Project for Medicine – learning outcomes for undergraduate medical education in Europe. Med Teach.

[5] Ross MT, Nikolić N, Peeraer G, Murt A, Kroiča J, Elcin M, Hope D, Cumming AD (2014). Report of the MEDINE2 Bachelor of Medicine (Bologna First Cycle) tuning project. Med Teach.

[6] Plat E, Scherer M, Bottema B, Chenot JF (2007). Facharztweiterbildung Allgemeinmedizin in den Niederlanden – ein Modell für Deutschland? Beschreibung der Weiterbildung und kritischer Vergleich. Gesundheitswesen.

[7] Bundesärztekammer (2018/2022). (Muster-)Weiterbildungsordnung 2018.

[8] Ten Cate O (2017). Competency-Based Postgraduate Medical Education: Past, Present and Future. GMS J Med Educ.

[9] McGaghie WC, Miller GE, Sajid AW, Telder TV (1978). Competency-based curriculum development on medical education: an introduction. Public Health Pap.

[10] Hartmannbund (2019). Wie gut können wir gute Ärzte werden? HB-Assistenzarztumfrage 2018/19.

[11] Hartmannbund (2021). Assistenzarzt-Umfrage 2021 – Arbeitsbedingungen, Ökonomisierung und Digitalisierung.

[12] Bundesärztekammer (2011). Ergebnisse der zweiten Befragung 2011.

[13] Kern DE, Thomas PA, Hughes MT (2016). Curriculum development for medical education: a six-step approach.

[14] Ende J, Atkins E (1992). Conceptualizing curriculum for graduate medical education. Acad Med.

[15] Yudkowsky R, Tekian A (1998). A model workshop in curriculum development for international medical audiences. Med Teach.

[16] Lindvall M, Basili V, Boehm B, Costa P, Dangle K, Shull F, Tesoriero R, Williams L, Zelkowitz M, Wells D, Williams L (2002). Empirical Findings in Agile Methods.

[17] Odzaly EE, Greer D, Stewart D (2018). Agile risk management using software agents. J Ambient Intell Humaniz Comput.

[18] Moran A (2014). Agile Risk Management.

[19] Mc Hugh M, McCaffery F, Casey V (2012). Barriers to using agile software development practices within the medical device industry.. EuroSPI.

[20] Beck K, Beedle M, Bennekum Av, Cockburn A, Cunningham W, Fowler M, Grenning J, Highsmith J, Hunt A, Jeffries R, Kern J, Marick B, Martin RC, Mellor S, Schwaber K, Sutherland J, Thomas D Manifesto for Agile Software Development.

[21] Schwaber K (2004). Agile project management with Scrum.

[22] Abrahamsson P, Salo O, Ronkainen J, Warsta J (2017). Agile software development methods: Review and analysis.

[23] Cockburn A, Highsmith J (2001). Agile software development: The people factor. Computer.

[24] Hidalgo ES (2019). Adapting the scrum framework for agile project management in science: case study of a distributed research initiative. Heliyon.

[25] Masood ZA, Farooq S (2017). The Benefits and Key Challenges of Agile Project Management under Recent Research Opportunities. Int Res J Manage Sci.

[26] Muhammad U, Nazir T, Muhammad N, Maqsoom A, Nawab S, Fatima ST, Shafi K, Butt FS (2021). Impact of agile management on project performance: Evidence from I.T sector of Pakistan. PLoS One.

[27] de Oliveira Santos P, de Carvalho MM (2022). Exploring the challenges and benefits for scaling agile project management to large projects: a review. Requir Eng.

[28] Griffin L, Hinek A (2022). An Analysis of Agile Coaching Competency Among Practitioners.

[29] Stray V, Memon B, Paruch L (2020). A systematic literature review on agile coaching and the role of the agile coach. Product-Focused Software Process Improvement: 21st International Conference, PROFES 2020, Turin, Italy, November 25–27, 2020, Proceedings 21; 2020.

[30] Ärztekammer Hamburg (2020). Weiterbildungsordnung der Hamburger Ärztinnen und Ärzte vom 15. Juni 2020.

[31] Union Européenne des Médicins Spécialists (2015). Training Requirements for the Specialty of Paediatrics. European Standards of Postgraduate Medical Specialist Training (old chapter 6).

[32] Cook DA (2010). Twelve tips for evaluating educational programs. Med Teach.

[33] Durning SJ, Hemmer P, Pangaro LN (2007). The structure of program evaluation: an approach for evaluating a course, clerkship, or components of a residency or fellowship training program. Teach Learn Med.

[34] Haji F, Morin MP, Parker K (2013). Rethinking programme evaluation in health professions education: beyond 'did it work?'. Med Educ.

[35] Barajaz M, Turner T (2016). Starting a new residency program: a step-by-step guide for institutions, hospitals, and program directors. Med Educ Online.

[36] Sierocinski E, Mathias L, Freyer Martins Pereira J, Chenot JF (2022). Postgraduate medical training in Germany: A narrative review. GMS J Med Educ.

[37] Fullan M The NEW meaning of educational change.

[38] Bano M, Zowghi D (2013). User involvement in software development and system success: a systematic literature review. Proceedings of the 17th International Conference on Evaluation and Assessment in Software Engineering.

[39] Branch R, Dousay T (2015). Survey of instructional design models.

[40] Stefaniak J, Xu MM (2020). An Examination of the Systemic Reach of Instructional Design Models: a Systematic Review. TechTrends.

[41] Gibbons AS (2013). An architectural approach to instructional design.

